# Altered Resting-State fMRI Signals in Acute Stroke Patients with Ischemic Penumbra

**DOI:** 10.1371/journal.pone.0105117

**Published:** 2014-08-14

**Authors:** Yuan-Hsiung Tsai, Rui Yuan, Yen-Chu Huang, Hsu-Huei Weng, Mei-Yu Yeh, Ching-Po Lin, Bharat B. Biswal

**Affiliations:** 1 Department of Diagnostic Radiology, Chang Gung Memorial Hospital at Chiayi, College of Medicine and School of Medical Technology, Chang-Gung University, Taoyuan, Taiwan; 2 Department of Biomedical Engineering, New Jersey Institute of Technology, Newark, New Jersey, United States of America; 3 Department of Neurology, Chang Gung Memorial Hospital at Chiayi, College of Medicine and School of Medical Technology, Chang-Gung University, Taoyuan, Taiwan; 4 Department of Biomedical Imaging and Radiological Sciences, National Yang Ming University, Taipei, Taiwan; 5 Institute of Neuroscience, National Yang Ming University, Taipei, Taiwan; National University of Singapore, Singapore

## Abstract

**Background:**

Identifying the ischemic penumbra in acute stroke subjects is important for the clinical decision making process. The aim of this study was to use resting-state functional magnetic resonance singal (fMRI) to investigate the change in the amplitude of low-frequency fluctuations (ALFF) of these subjects in three different subsections of acute stroke regions: the infarct core tissue, the penumbra tissue, and the normal brain tissue. Another aim of this study was to test the feasilbility of consistently detecting the penumbra region of the brain through ALFF analysis.

**Methods:**

Sixteen subjects with first-ever acute ischemic stroke were scanned within 27 hours of the onset of stroke using magnetic resonance imaging. The core of infarct regions and penumbra regions were determined by diffusion and perfusion-weighted imaging respectively. The ALFF were measured from resting-state blood oxygen level dependent (BOLD) fMRI scans. The averaged relative ALFF value of each regions were correlated with the time after the onset of stroke.

**Results:**

Relative ALFF values were significantly different in the infarct core tissue, penumbra tissue and normal brain tissue. The locations of lesions in the ALFF maps did not match perfectly with diffusion and perfusion-weighted imagings; however, these maps provide a contrast that can be used to differentiate between penumbra brain tissue and normal brain tissue. Significant correlations between time after stroke onset and the relative ALFF values were present in the penumbra tissue but not in the infarct core and normal brain tissue.

**Conclusion:**

Preliminary results from this study suggest that the ALFF reflects the underlying neurovascular activity and has a great potential to estimate the brain tissue viability after ischemia. Results also show that the ALFF may contribute to acute stroke imaging for thrombolytic or neuroprotective therapies.

## Introduction

Recent progress in the acute management of ischemic stroke subjects has shown that thrombolysis treatment increases survival and reduces disability of the subjects [1], [2]. The ischemic penumbra, which is the target tissue for thrombolysis and reperfusion, is still viable but hypoperfused and at risk of becoming infarct. The term “penumbra”, originally introduced by Astrup and colleagues, is classically defined as the hypoperfused tissue surrounding the ischemic core in which blood flow is too low to maintain electrical activity but sufficient enough to preserve ion channels [3]. Neurons in penumbra area still retain enough energy to sustain resting membrane potentials [4]. When reperfusion or collateral circulation is established, the action potentials will be restored and these areas will recover. Without reperfusion, the brain cells in penumbra will die, and the lesion will expand. Clinically, the therapeutic time window for thrombolysis is within hours after the stroke [2], [5]. However, it is likely that stroke subjects may benefit from thrombolysis and reperfusion procedures after this time window and exhibit heterogeneous recovery [5]. There has been a signifcant rise in the number of studies using neuroimaging to extend and assess the therapeutic window and the effect of thrombolytic therapy. In the penumbra region, there are reductions in the cerebral blood flow and increases in the oxygen extraction fraction and the cerebral metabolic rate of oxygen consumption. These changes in regional perfusion and metabolic measures, although highly variable, have been well documented using positron emission tomography and MRI [6]–[8]. Clinically, the mismatch between the diffusion weighted image (DWI) and perfusion weighted image (PWI) is widely used to detect the penumbra tissue.[9], [10]. However, this method has limitations for detecting the penumbra tissue [6], and the mismatch of PWI/DWI provides only an approximation of the penumbra [8], [11], [12]. PWI directly measures cerebral blood flow but not oxygen metabolism and neuron metabolism, and therefore, does not reflect the disease’s pathophysiology accurately [8]. New imaging biomarkers, which improve the assessment of the viability of brain tissue, can augment existing penumbral imaging and influence decision making for thrombolytic or neuroprotective therapy [13].

Functional MRI has been used to measure changes in BOLD signals reflecting hemodynamic response, which is highly correlated with neural activities. In recent years, resting-state functional connectivity, using BOLD fMRI signal changes, have been used to demonstrate spatiotemporal correlations within functional networks [14]. Synchronized low frequency fluctuations (LFFs) of BOLD signal have been successfully employed in many studies to investigate resting-state networks involved in primary motor and primary sensory networks, language networks [15], [16] as well as altered networks in clinical populations including schizophrenia, attention deficit hyperactivity disorder, major depression, and Alzheimer’s diseases [17]–[20]. Furthermore, the amplitude of low-frequency fluctuation (ALFF) of the resting-state BOLD signal measure has been shown to reflect spontaneous neural and cerebrovascular reactivity [21]–[23]. A previous work by Li et al. showed that resting BOLD fMRI-derived measures, including ALFF and regional homogeneity (ReHo, used to present the level of homogeneity or association of regional voxels), are coupled with regional CBF and are therefore linked to regional spontaneous brain activity [22]. We have previously shown that ALFF correlates significantly with hypercapnic fMRI response in healthy subjects [24], [25] which suggests that the ALFF reflects the underlying hemodynamic function.

Currently, there have been two fMRI studies in animal models that have measured the ALFF in acute stroke subjects. Liu and colleagues reported decreased frequency and increased magnitude of ALFF in the peri-infarct tissue in nonhuman primate stroke model [26]. A study done by Yao and colleagues demonstrated dynamic changes in the ALFF in super-acute stroke rat model with middle cerebral artery occlusion [27]. The magnitude of abnormal ALFF increased and migrated from the ischemic core to the peripheral edge to exceed the borderline of the DWI within 24 hours after middle cerebral artery occlusion. These animal studies suggested that the measurement of the ALFF may have potential for estimating and predicting viability and prognosis of acute ischemic brain tissue. To the best of our knowledge, a voxel wise measure of the amplitude of the resting-state fluctuations has not been conducted in human stroke patients. Therefore, the goal of our current study was to investigate the change in the ALFF in brain tissue after the onset of cerebral ischemia. Several other measurements, including fractional ALFF (fALFF), ReHo and standard deviation (StDev) maps were also estimated from the resting-state BOLD signal. We hypothesized that change in the ALFF after stroke is associated with the decline of neurovascular viability and is different in the infarct core tissue, penumbra tissue and normal brain tissue. We also hypothesize that ALFF changes and differed in each time point after stroke onset. the amplitude and frequency of LFFs should occur in ischemic regions.

## Materials and Methods

### Subjects

Sixteen first-ever acute ischemic stroke patients were enrolled. All of the following conditions were used to exclude a subject from the study: Subjects with hyperacute stroke within three hours after onset that are indicated for thrombolytic therapy; patients with unclear time of stroke onset; patients with history of previous neurological insult or psychiatric disorder, and any abnormality except for the acute infarction was found in conventional diagnostic MRI.

The existence of the penumbra in each patient was determined by the target mismatch profile Tmax (time-to-maximum) >6 sec lesion volume/DWI lesion volume >1.2 and absolute difference between Tmax >6 sec lesion volume and DWI lesion volume >10 mL [5]. The National Institutes of Health Stroke Scale (NIHSS) and duration after stroke onset to MRI scan were recorded before MRI scan. The study was part of an integrated stroke project at Chang Gung Memorial Hospital and was approved by the Institutional Review Board of Chang Gung Memorial Hospital. All patients, or their families, gave their written informed consent prior to participation in the study.

### MRI instrumentation and procedures

All data were collected using a 3 Tesla Siemens Vario MRI system (Siemens Medical System, Erlangen, Germany) with a 32 channel head coil. The imaging parameters for each subject was as follows: T1-weighted (T1WI) anatomical images were obtained with a gradient echo sequence (TR = 3500 ms, TE = 2.87 ms, FOV = 220 mm, matrix = 256×256, 160×1-mm slices, resulting in a spatial resolution of 0.9 mm×0.9 mm×1.0 mm, scan time = 7 minutes 11 seconds). DWI were acquired using a single-shot spin-echo diffusion-sensitized EPI pulse sequence (TR = 5600 ms, TE = 93 ms, FOV = 230 mm, matrix = 130×130, 28×4-mm slices, scan time = 1 minutes 2 seconds). Three diffusion-weighted images with diffusion sensitivity (b_1_ = 1000 s/mm^2^) were applied sequentially in the x, y, and z gradient directions and a reference image without diffusion sensitivity (b_0_≈0 s/mm^2^) was acquired. Dynamic susceptibility contrast perfusion weighted imaging scans were acquired by using a gradient echo EPI (TR = 1500 ms, TE = 36 ms, FOV = 220 mm, matrix = 92×92, 17×6-mm slices, scan time = 1 minutes 36 seconds) with an intravenous bolus injection of gadolinium contrast agent (0.2 mmol/Kg) at the 5^th^ dynamic. During resting-state BOLD fMRI scans, subjects were instructed to stay awake and relaxed with their eyes closed. A gradient echo EPI, sensitive to BOLD contrast, was used (TR = 2500 ms, TE = 27 ms, FOV = 220 mm, matrix = 64×64, 36×4-mm slices). Each scan consisted of 240 image volumes and lasted for 10 minutes and 7 seconds.

### Perfusion Data Preprocessing

Dynamic susceptibility contrast weighted images were analyzed with perfusion mismatch analyzer software (version: 3.4.0.7 Jun 2011; http://asist.umin.jp/index-e.htm). Time-to-maximum (Tmax) maps were generated to demonstrate the penumbra area.

### Functional Data Preprocessing

The fMRI data processing was accomplished using Analysis of Functional NeuroImages (AFNI) software (http://afni.nimh.nih.gov/afni; version: Jan 2012) [28] and FMRIB Software Library (FSL) (http://www.fmrib.ox.ac.uk/fsl/; version: 4.1.8). The first 5 time points were removed from all the time series. AFNI command ‘3dvolreg’ was used to perform head motion correction on all datasets. Functional images were coregistered to the subject’s anatomical image. The anatomical images were segmented into the gray matter, the white matter and the cerebral spinal fluid (CSF). Six motion parameters, the Euclidean norm of motion derivatives, and the 5 principle components from both CSF and white matter have been regressed out from all the time series for each subject. All data and related metadata must be deposited in an appropriate public repository, unless already provided as part of the submitted article or supporting information. If there are restrictions on the ability of authors to publicly share data–e.g. privacy or use of data from a third party– these reasons must be specified.

#### ALFF and fractional ALFF map

The time series of resting-state fMRI data of each voxel was linearly detrended. ALFF and fALFF were calculated by using the Resting-State fMRI Data Analysis Toolkit V1.7 (REST) (http://www.restfmri.net [date last accessed 25 May 2012]). For each voxel, discrete Fourier transform was performed on resting-state time series. The cumulative power spectrum between 0.01 and 0.08 Hz in each voxel was estimated as the ALFF value. The proportion of the ALFF value over the cumulative power of all frequencies (below the Nyquist’s frequency) is the fALFF value.

#### ReHo Map

The time series of resting-state fMRI data of each voxel was linearly detrended and filtered (0.01 to 0.08 Hz). ReHo was performed in a voxelwise manner by calculating Kendall’s coefficient of concordance of time series of a given cluster to the neighboring voxels [29]. The cubic cluster size was 27 voxels and the central voxel of every cubic cluster was assigned a ReHo value. A large ReHo value for a given voxel indicates a higher local synchronization of resting-state fMRI signal among neighboring voxels.

#### Standard Deviation maps

The standard deviation of the time series at each voxel represents the different levels of variance of the BOLD signal from the infarct regions and in the penumbra regions. The mean value of each voxel was also calculated. This parameter provides information about the baseline of neuronal activities at different regions.

#### Z-score map

In order to eliminate extreme values and increase the contrast, z-score transform (−2.3<z<2.3) was used to enhance the contrast of ALFF, fALFF and ReHo maps. Thus, those ALFF, fALFF, ReHo voxels are standardized by subtracting its global mean and dividing by its standard deviation Z score was computed for every voxel. Values ranging between −2.3 to 2.3, which present nearly 99% of all values, were left untouched, while those outside the range were modified. All values under −2.3 are set to −2.3 and all values above 2.3 are set to 2.3. Thus, all values in ALFF, fALFF and ReHo were fit into the range between −2.3 to 2.3.

### Regions of interest

**Figure 1:**
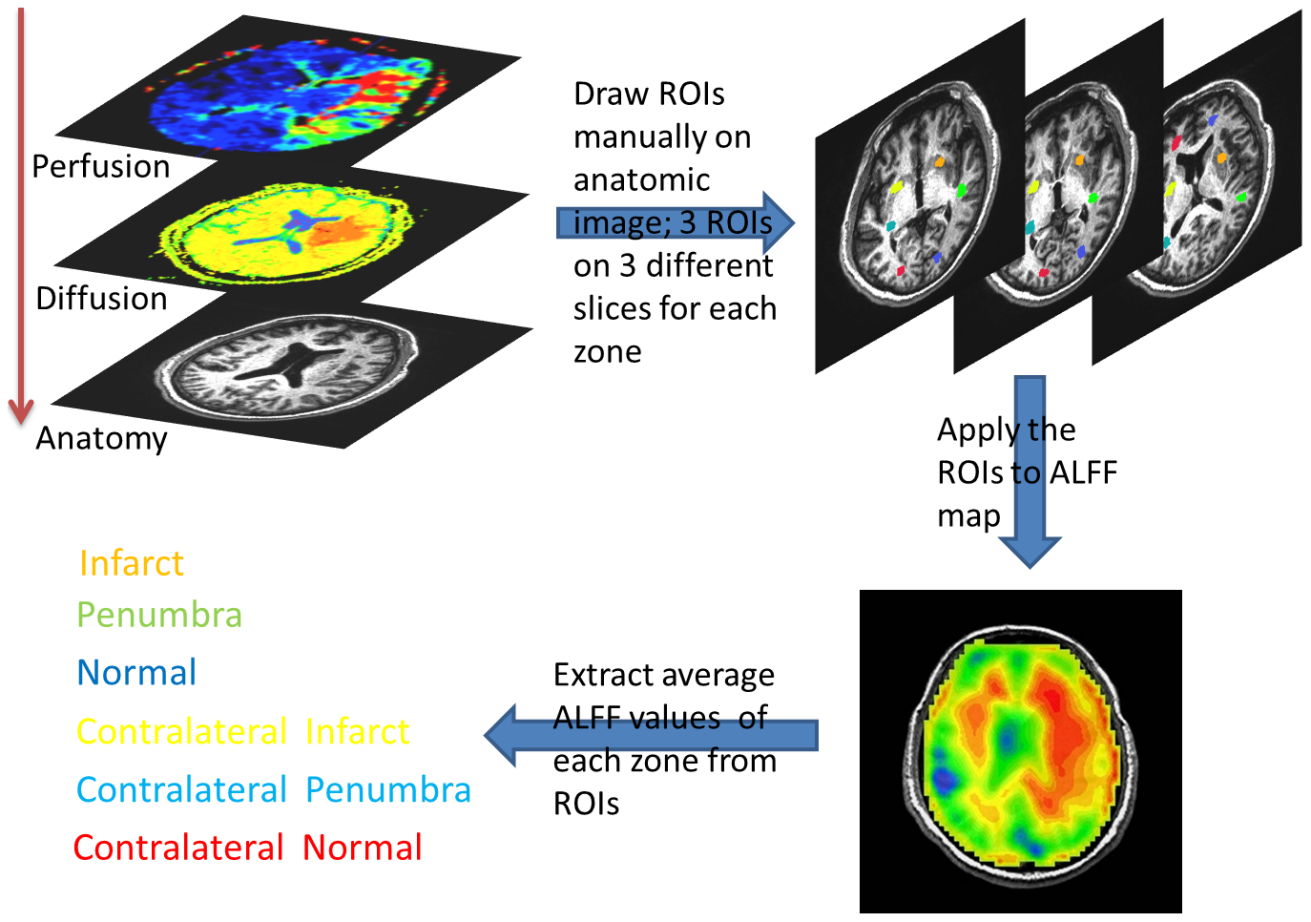
Flow diagram for ROI selection. The T1WI anatomic imaging, ADC and Tmax maps were displayed simultaneously when selecting the ROIs manually on anatomic T1WI imaging. Three ROIs were drawn at different slices for each area. The ROIs were than applied to the resting-state BOLD EPI map to get the ALFF value.

For quantitative analysis of the ALFF, the ROIs masks for each subject were manually circled by a neuroradiologist (T.Y.H). The steps used for selecting ROIs are illustrated in figure 1. While selecting ROIs on T1 anatomical imaging, the ADC and Tmax maps were displayed simultaneously for precise comparison. The area of penumbra was defined by the threshold of Tmax >6 seconds, excluding infarct core defined by ADC. For subjects with penumbra, the ROIs masks included infarct, penumbra, and ipsilateral normal regions as well as the corresponding contralateral brain regions on the contralateral normal tissue. For subjects without defined penumbra, only infarct, ipsilateral normal and corresponding contralateral regions were selected. Three ROIs were drawn at different slices for each area avoiding large cerebral spinal fluid spaces. The ROIs were than applied to the resting-state BOLD EPI map to get the ALFF value. For each area, the amplitude of ALFF was defined as the average of ALFF values in each voxel included in all three ROI masks. The relative ALFF for each area on the lesion side was calculated by taking the ratio of ALFF on the lesion side and ALFF on the contralateral normal side.

### Statistics

One-way ANOVA was performed to evaluate the relative ALFF differences between infarct core, penumbra and normal brain tissue of the 12 patients with pernumbra. Post Hoc multiple comparisons were performed by Fisher’s Least Significant Difference (LSD) method. Analysis of correlation between time after stroke onset and the relative ALFF in each area was performed with bivariate correlations. The correlations were presented with Spearmanrank correlation coefficients. A P value less than 0.05 was deemed a significant correlation. All statistical analyses were performed using Statistical Product and Service Solutions (SPSS, Version 18,2010).

## Results

Sixteen subjects were used in analysis (mean age: 71.1±14.5 years; range: 36–89 years; 7 male and 9 female; all right-handed). The demographic data of the patients are listed in Table 1. The mean time interval between stroke onset to MRI scan is 12.2±7.3 hours (range: 3.1–27.0 hours). Penumbra areas defined by the Target mismatch profile were presented in 12 patients out of a total of 16 patients.

**Table 1:** Demographic Data and Relative ALFF value of the stroke subjects.

Subject	Age	Gender	Time to MRI (h)	Penumbra	NIHSS	Relative ALFF		
						Infarct	Penumbra	Normal
1	81	F	24	+	23	0.85	0.88	0.94
2	72	M	27	−	2	0.79		1.04
3	86	F	17.2	−	7	1.06		1.06
4	83	F	16.8	−	4	0.83		1.16
5	85	F	3.1	+	2	0.75	1.20	1.04
6	71	M	14.5	+	8	0.80	0.79	1.22
7	86	F	9.5	+	13	0.72	0.75	1.03
8	60	F	12.3	−	1	0.92		2.89
9	62	M	3.2	+	17	0.75	0.86	1.23
10	89	F	3.8	+	21	0.66	1.29	0.99
11	36	M	5.6	+	18	0.89	1.18	1.15
12	71	M	7.5	+	5	1.00	0.95	1.02
13	71	F	16	+	7	0.90	0.89	1.10
14	58	F	9.6	+	7	1.00	1.20	1.01
15	53	M	17.8	+	15	0.95	0.95	1.06
16	73	M	6.6	+	6	0.97	0.93	1.16

M = male; F = female; h = hour; ALFF = Amplitude of Low-Frequency Fluctuation; NIHSS = National Institutes of Health Stroke Scale.

### fMRI results

**Figure 2:**
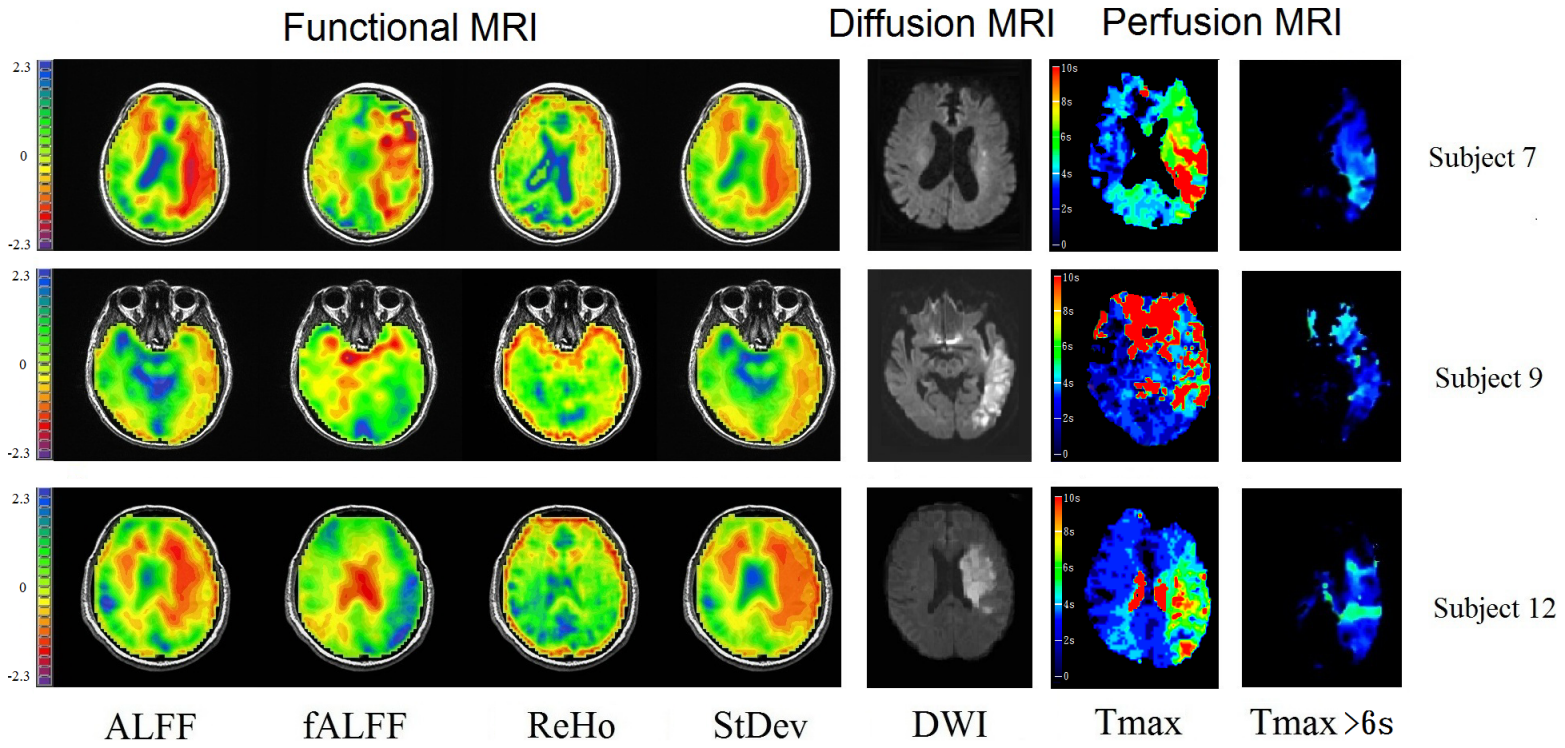
Illustrative resting fMRI, DWI and Tmax maps in three cases. The bright regions in DWI represented core of infarction and the defects in Tmax maps include both infarct core and penumbra area. ALFF indicates amplitude of low frequency fluctuation; fALFF: fractional ALFF; ReHo: regional homogeneity; StDev: Standard Deviation maps; DWI: diffusion-weighted image; Tmax: time-to-maximum; s: second. The scale bars for the fMRI represent Z score.

Figure 2 illustrates fMRI, DWI, MTT and Tmax maps of 3 acute stroke patients. The fMRI data of ALFF, fALFF, ReHo and StDev were all z-scored. The regions of DWI lesions represent infarct cores while the areas between perfusion defects and DWI lesions represent penumbra zones. Regions outside the penumbra areas are normal or benign oligemia areas (normal or mild decrease of perfusion and the tissue is not at risk for developing into infarction). Although the locations of the lesion did not match completely with perfusion maps, the ALFF, fALFF and StDev maps provided contrast to distinguish the infarct and penumbra regions from normal brain regions.

The relative ALFF values in each area are shown on the right column in Table 1. In general, the ALFF in the infarct core is comparatively smaller than the ALFF in the contra-lateral corresponding areas for 13 out of 16 subjects. The ALFF in the normal or benign oligemia areas is relatively larger than the ALFF in of the contra-lateral corresponding areas for 14 out of 16 subjects. The ALFF in the penumbra area does not show any obvious trend. The result of one way ANOVA test showed significant differences in the relative ALFF values between infract, penumbra and normal regions (p = 0.001). Significant difference of relative ALFF values between infarct and normal (p<0.001; 95%CI: 0.1151∼0.3354) as well as infarct and penumbra (p = 0.0088; 95%CI: 0.0420∼0.2623) were confirmed by multiple comparisons.

### Correlation between stroke onset duration and relative ALFF

**Figure 3:**
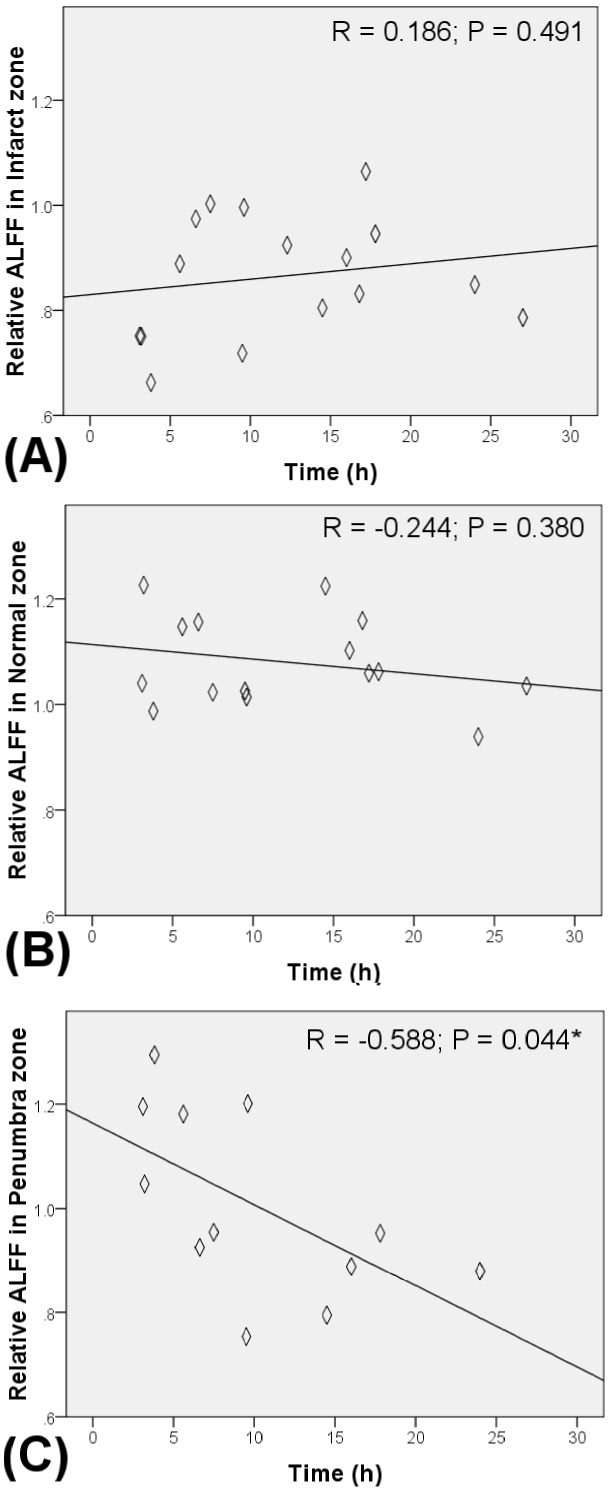
Scatter plots showing the relationships between relative ALFF and time after stroke in (A) Infarct, (B) Ipsilateral normal and (C) Penumbra zones.

In order to clarify the relashionship between ALFF and duration of stroke onset, the correlations between stroke onset duration and the relative ALFF value of penumbra, infarct and normal regions were calculated. Significant negative correlations between the time interval of stroke onset to MRI and the relatively ALFF was presented in the penumbra regions (R = −0.588, P = 0.044, Figure 3C). On the contrary, the relative ALFF value of the infarct core tissue and ipsilateral normal tissue was not significantly correlated with the time interval (Figure 3A and 3B). The relative ALFF values in both normal tissue and infarct tissue exhibited significantly smaller variation than the relative ALFF values from penumbra regions. In addition, relative ALFF values in penumbra regions decrease over time. During the first 5 hours after the onset of stroke, the level of relative ALFF values at penumbra regions is close to the relative ALFF values in normal brain tissue. After 15 hours of stroke onset, the level of relative ALFF values in penumbra tissue decreased to the ALFF value similar to the infarct tissue.

## Discussion

The hemodynamic statuses of brain after stroke differ from patient to patient and depend on their physiological status, medications, level of artery occlusion as well as early development of collateral circulation. Most of them affect not only the ischemic area, but also the ipsilateral/contralateral healthy brain. Thus the contralateral brain tissue is not exactly normal, and this makes it difficult to use the absolute ALFF value in each brain area for quantitative measurement. In this study, we used the relative ALFF value in order to reduce the effect of underlying heterogeneous physiology. The core of the infarct brain tissue generally showed decreased relative ALFF values, which were not correlated with the duration after stroke onset. This result is consistent with the previous work by Liu et al. in nonhuman primate stroke model [26]. Brain tissues in the infarct core region, defined here with DWI, suffer from cytotoxic swelling, membrane ion pump failure and irreversible cell death [30]. The blood flow markedly decreases while the oxygen consumption and neuronal activity vanishes. These physiological responses contribute to a steady and low relative ALFF value. The ALFF values in ipsilateral normal brain tissue were generally higher than in the contralateral corresponding area. This indicates that an increase in microvessel density [31], dilation of blood vessels, or compensatory neural activities may increase the regional ALFF. The relative ALFF values in penumbra area, however, varied from subject to subject. As shown in Table 1, the relative ALFF values in penumbra area were not strictly higher or lower than 1. This is different from the results of Liu et al., who found an increased magnitude in the ALFF in the peri-infarct tissue. The relative ALFF values in penumbra area were negatively correlated with the durations after stroke onset. As time progresses, the relative ALFF values in penumbra region decrease. This may imply that the relative ALFF values in penumbra area change from those in normal to infarct area with time. Brain tissue in penumbra area was found to maintain sufficient metabolism to sustain cell membrane potentials [5], [13], [32]. The blood flow and oxygen consumption rate in penumbra area, although significantly decreased, are preserved to maintain the survival of neuronal cells. In addition, the neurovascular units, which include neurons, glia and endothelial cells, may activate to uphold neuroprotective function or generate cell death [32], [33]. The fluctuations of BOLD signal shouldbe affected by both neural and vascular activities. Several recent publications have focused on seperating the BOLD based signal effects from vascular effects [22]. In studies where the neurovascular activities may have been compromised, it would be useful to have simultaneous measurements of vascular and neural factors. Without reperfusion, these neurovascular activities dissolve and the ALFF value decline. Based on these observations, detection of penumbra by ALFF maps has potential to provide additional information regarding neurovascular activities in penumbra area.

In addition to the ALFF maps, which represent the power of low frequency fluctuations in the BOLD signal, we demonstrated three other types of fMRI map: fALFF, ReHo, StDev. Although ALFF is effective at detecting low frequency fluctuations, the fluctuations detected can extend over 0.1 Hz, particularly near major vessels [23] that are characterized by widespread oscillations across both low and high frequencies. In contrast, the fALFF is defined as the total power within the low-frequency range (0.01–0.1 Hz) divided by the total power in the entire detectable frequency range, which is determined by the sampling rate and duration. Despite the differences in sensitivity to artifacts, the ALFF and fALFF exhibited regional differences within the penumbra area. Since neurons in the same region might share similar behaviors, ReHO is used to present these different regions by the level of homogeneity or association of voxels. The level of homogeneity or similarity of each voxel is measured by Kendall’s Coefficient of Concordance (KCC). KCC calculates the rank correlation between the time series of each voxel and its neighboring voxels. This results in a more homogeneous map but provides less imaging contrast than the ALFF map. We also calculated the standard deviation of the time series at each voxel. The standard deviation map demonstrated variations of the time series in each voxel. Since the infarct regions have less neuronal activities than penumbra regions, time points of time series in infarct regions may have less deviation from the baseline than these in penumbra regions.

This study, being preliminary in nature, has several limitations. First, our sample size consisted of only 16 subjects, which is relatively small. Since MRI scanning may delay intravenous thrombolytic therapy, we excluded patients who suffered from a hyperacute stroke within 3 hours after the onset of stroke and were indicated for intravenous thrombolytic therapy. Since the pathophysiology of penumbra tissue changes rapidly during hyperacute stage, the ALFF changes during this stage may be dramatic and should provide more information about the underlying mechanism. Also, a longitudinal imaging study to see the dynamic change of the ALFF in each individual stroke patient will help researchers and clinicians understand how the ALFF changes over time and how it relates to the pathophysiology. Another limitation was the presence of motion and ghosting in some subjects. It is difficult for acute stroke subjects to remain still in the MRI. We excluded subjects who had too much ghosting and head motion after preprocessing. A number of subjects still displayed a minor degree of ghosting and head motion even after spatial realignment.

## Conclusions

In this study, we demonstrated the alterations of ALFF values in infarct core, penumbra and normal brain tissue in acute infarction patients. The relative ALFF values of penumbra area were correlated with the time after stroke onset. These findings suggest that ALFF maps derived from resting-state fMRI have the potential to estimate brain tissue viability after ischemia. ALFF maps may be able to provide an imaging contrast to differentiate the infarct core tissue, penumbra tissue and normal brain tissue. This would aid in the clinical decision making processes related to thrombolytic and neuroprotective therapies.
